# An Analog Multilayer Perceptron Neural Network for a Portable Electronic Nose

**DOI:** 10.3390/s130100193

**Published:** 2012-12-24

**Authors:** Chih-Heng Pan, Hung-Yi Hsieh, Kea-Tiong Tang

**Affiliations:** Department of Electrical Engineering, National Tsing Hua University, No. 101, Sec. 2, Kuang-Fu Road, 30013 Hsinchu, Taiwan; E-Mails: u931814@oz.nthu.edu.tw (C.-H.P.); hyhsieh@larc.ee.nthu.edu.tw (H.-Y.H.)

**Keywords:** analog MLP circuit, electronic nose

## Abstract

This study examines an analog circuit comprising a multilayer perceptron neural network (MLPNN). This study proposes a low-power and small-area analog MLP circuit to implement in an E-nose as a classifier, such that the E-nose would be relatively small, power-efficient, and portable. The analog MLP circuit had only four input neurons, four hidden neurons, and one output neuron. The circuit was designed and fabricated using a 0.18 μm standard CMOS process with a 1.8 V supply. The power consumption was 0.553 mW, and the area was approximately 1.36 × 1.36 mm^2^. The chip measurements showed that this MLPNN successfully identified the fruit odors of bananas, lemons, and lychees with 91.7% accuracy.

## 1. Introduction

The artificial olfactory system, also referred to as the electronic nose (E-nose) system, has been used in numerous applications. These include air quality monitoring, food quality control [1], hazardous gas detection, medical treatment and health care [2], and diagnostics [3]. An E-nose system comprises a sensor array, a signal processing unit, and a pattern recognition system. During recent decades a substantial amount of research and development has been reported on E-nose systems. Because of the complex classification algorithms embedded in the pattern recognition system, a central processing unit (CPU) is usually required [1,2]. Consequently, the majority of E-nose systems is large and consumes considerable power. However, heavy and power-hungry equipment is inconvenient to use, and designing a low-power small device would be preferable. Some researchers and companies [4–6] have used microprocessors or field-programmable gate arrays (FPGAs) as a computational cell to develop portable E-noses, but these systems are still too power-intensive and large. To further reduce the power consumption and device area, analog VLSI implementation of the learning algorithm for E-nose application has been proposed [7–14].

The multilayer perceptron neural network (MLPNN) is an algorithm that has been continuously developed for many years. Consequently, when VLSI implementation of a learning algorithm is necessary, MLPNN is a common choice. In 1986, Hopfield and Tank proposed the first analog MLPNN circuit [10]. Since then, several analog VLSI implementations of MLPNN have been proposed [11–14]. Some have focused on the improvement of the multiplier [14–18] and some have attempted to design a nonlinear synapse to remove the analog multiplier [19,20]. However, the power consumption for most of the MLPNN circuits range from a few milliwatts to a few hundred milliwatts [11–14]. This power consumption is still too high for portable applications. Consequently, an MLPNN circuit with considerably lower power consumption (lower than 1 mW) is required when an MLPNN is being implemented in a portable E-nose.

This study implemented a low power MLPNN by analog VLSI circuit to serve as a classification unit in an E-nose. Neural networks is one of the most popular algorithms used in an E-nose system [2,5], because it can recognize and identify odor signal patterns. A typical MLPNN contains one input layer; one or more hidden layer(s), depending on the application; and an output layer. Apart from the input layer, both the hidden and output layers contain several neurons with nonlinear activation functions, which constitute the signal processing unit. Synapses connect the neurons of different layers, and a weight unit is included in each synapse. The weight units and the outputs of neurons determine the input of neurons in subsequent layers. An analog circuit realizes a nonlinear function with a simple structure [15]. Thus, implementing an analog MLPNN circuit reduces the need for power and the size of the pattern recognition unit required to build an E-nose.

The weight adaptation algorithm used in this study was the back propagation (BP) learning algorithm [21]. This algorithm allows the weights to be adjusted so that the MLP network can learn the target function; that is, pattern recognition. The details of the MLPNN and BP algorithms are provided below.

The input of a neuron can be represented as:

(1) $${\text{a}}_{j}^{H}=\sum_{i=1}^{n^{I}}W_{ji}^{H}\;{\text{X}}_{i}^{I}$$

where *a_j_^H^* is the input of the *j^th^* hidden neuron; *W_ji_^H^* represents the weight of the synapse that connects the *j^th^* hidden neuron and the *i^th^* input neuron; *X_i_^I^* represents the output of the *i^th^* input neuron; and *n^I^* is the number of input neurons. Neuron output is determined by its input and activation function. The hyper tangent function is one of the most commonly used activation functions. This function can be easily implemented by analog VLSI with small chip area and power consumption. Consequently, the hyper tangent activation was chosen for this work. By choosing hyper tangent activation function, the neuron output is:

(2) $${\text{X}}_{j}^{H}=\tan\;\text{h}({\text{a}}_{j}^{H})+b_{j}$$

where *X_j_^H^* is the output of the *j^th^* hidden neuron, and *b_j_* represents the bias value.

Similar to the hidden neuron, the input of the *k^th^* output neuron is:

(3) $${\text{a}}_{k}^{O}=\sum_{j=1}^{n^{H}}W_{kj}^{O}\;{\text{X}}_{j}^{H}$$

The output of the *k^th^* output neuron *X_k_^O^* is:

(4) $${\text{X}}_{k}^{O}=\tan\;\text{h}({\text{a}}_{k}^{O})+c_{k}$$

where *c_k_* represents the bias value.

After calculating the output *X_k_^O^* of the output neuron, we derived the adapting value by comparing the circuit output *X_k_^O^* with target output *X_t_*.

According to the BP algorithm, the general weight update value *ΔW* is derived by:

(5) $$\Delta \text{W}=-\eta \frac{\partial {\text{E}}_{\text{p}}}{\partial \text{a}}\frac{\partial \text{a}}{\partial W}$$

where *a* represents the neuron input; *η* is the learning rate, and *E_p_* is the error term derived from the comparison between circuit output *X_k_^O^* and target *X_t_*. In the following content, we used the mean square error and assumed that there was a single output neuron. Consequently, *X_k_^O^* was simplified to *X^O^*.

The order of weight adaptation proceeds from the output layer to the input layer. When deriving *ΔW* in each layer, repeatedly differentiating the error by weight is unnecessary because certain computations have already been performed in later layers (for the hidden layer, the later layer is the output layer). The later layer propagates the computation to the previous layer; this propagating value is called the “back propagation error.” For the output layer, the BP error *δ^O^* is:

(6) $$\delta^{O}=-\frac{\partial {\text{E}}_{\text{p}}}{\partial {\text{a}}^{O}}=(X_{t}-X^{O})\times D^{O}$$

where *D^O^* represents the differentiation of the output neuron's activation function. For the hidden layer, the BP error of the *j^th^* hidden neuron $\delta_{j}^{H}$ is:

(7) $$\delta_{j}^{H}=-\frac{\partial {\text{E}}_{\text{p}}}{\partial {\text{a}}_{j}^{H}}=\delta^{O}\times {\text{W}}_{j}^{O}\times {\text{D}}_{j}^{H}$$

From Equations (1), (3), (5), (6) and (7), *ΔW_j_^O^* and *ΔW_ji_^H^* are derived as Equations (8) and (9), respectively:

(8) $$\Delta {\text{W}}_{j}^{O}=-\eta \times (X_{t}-X^{O})\times D^{O}\times \frac{\partial {\text{a}}^{O}}{\partial {\text{W}}_{j}^{O}}=-\eta (X_{t}-X^{O})\times D^{O}\times {\text{X}}_{j}^{H}$$

(9) $$\Delta {\text{W}}_{ji}^{H}=-\eta \times (\delta^{O}\times {\text{W}}_{j}^{O}\times {\text{D}}_{j}^{H})\times \frac{\partial {\text{a}}_{j}^{H}}{\partial W_{ji}^{H}}=-\eta \times (\delta^{O}\times {\text{W}}_{j}^{O}\times {\text{D}}_{j}^{H})\times {\text{X}}_{i}^{I}$$

The MLPNN was trained by adapting the weights according to Equation (10). During the training phase, the new weight *W_new_* in a synapse was acquired from the previous weight *W_old_* in the same synapse plus the weight update value *ΔW*:

(10) $${\text{W}}_{\text{new}}=W_{\text{old}}+\Delta W$$

The rest of this paper is organized as follows: Section 2 describes the system architecture, Section 3 presents the measurement results, and Section 4 presents the conclusion.

## 2. Architecture and Implementation

**Figure 1.**
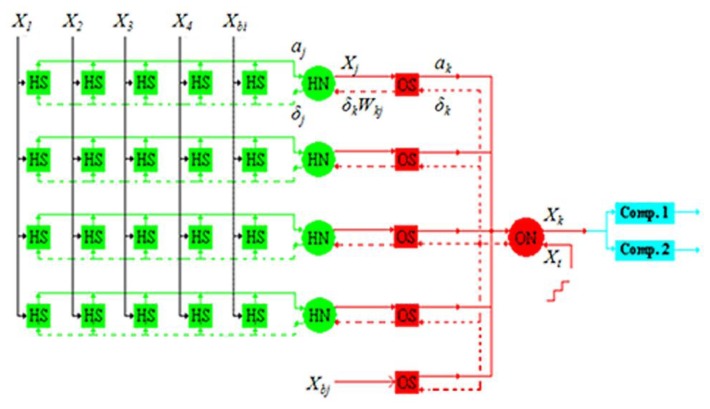
Block diagram of the proposed 4-4-1 MLPNN.

This paper proposes a 4-4-1 MLPNN. The 4-4-1 notation represents four input neurons, four hidden neurons, and one output neuron. This structure was proven by Matlab to be able to learn the odor data we used before really doing chip design. The block diagram is shown in Figure 1. The symbols *X_1_*–*X_4_* refer to four signal inputs; *X_bi_* is the bias in the input layer; *HS*s are the synapses between the input and hidden layers; *HN*s are the hidden neurons; *X_bj_* is the bias in the hidden layer; *OS*s are the synapses between the hidden and output layers; and *ON* is the output neuron.

**Figure 2.**
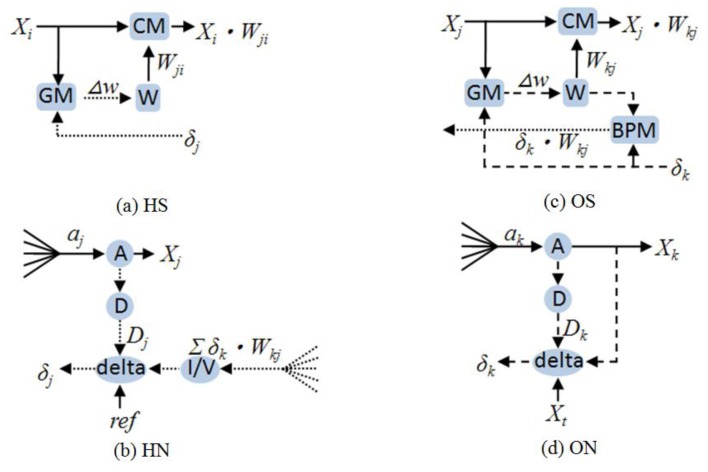
Detailed block diagrams of HS, HN, OS, and ON.

Based on Equations (1) to (10), the detailed block diagram of *HS*, *HN*, *OS*, and *ON* was obtained, as shown in Figure 2. The *CM, BPM* and *GM* are multipliers; *W* is weight; *A* is the activation function; *D* is the differentiation of activation function; *delta* is the BP error generator; and *I/V* is the current-to-voltage converter.

Because several multiplication results are summed in Equations (1) and (3), the output signals of all multipliers are designed as current signals. Using Kirchhoff's current laws (KCL), the current is summed if the outputs of the synapses are connected. Thus, the area and power of the system are reduced because no extra analog adder is necessary. For signals that require transmission to several nodes (e.g., X_1_ to X_4_), a voltage signal is preferred. Further description of the subblocks shown in Figure 2 and the relations between the equations and sub-blocks are provided below. When approximating the equations by analog circuit, second-order effects, such as the body or Early effect, are neglected; thus, particular errors may be introduced. These errors may result in nonlinearity. However, by carefully designing the bias, size, and dynamic range of the circuit, the nonlinearity has little effect on the learning performance of the application in this study.

### 2.1. Synapses

**Figure 3.**
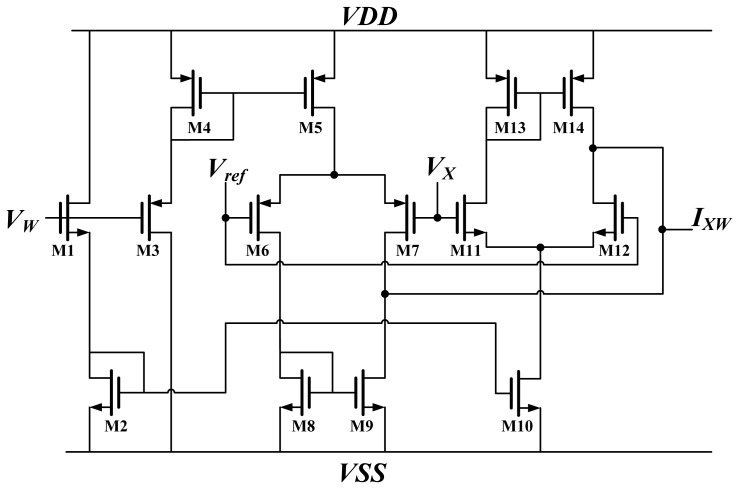
Schematic of Chible's multiplier.

The synapses at the hidden layer and the output layer comprise two multipliers (*CM* and *GM*) and a weight unit *W*; the output layer synapse needs one more multiplier (*BPM*) to generate the term *δ^O^_kk_W_j_^O^*. The terms *BPM* and *CM* are both Chible's multipliers [16,17]. The *BPM* multiplies the weight by BP error, whereas *CM* is used to multiply the input by weight. The results represent the current. This study used the Chible's multiplier because of its wide operation range [16]. A schematic diagram of Chible's multiplier is shown in Figure 3.

The weight and input are voltages, denoted by *V_W_* and *V_X_*, respectively. The output of the multiplier is *I_XW_*. M1, M3 operate in strong inversion regions, whereas M6, M7, M11, and M12 operate in weak inversion regions. According to the equation of MOSFET in strong and weak inversion, the output current is:

(11) $${\text{I}}_{WX}=(I_{wp}-I_{wn})\times \tan\;\text{h}\left(\frac{\kappa (V_{x}-V_{\text{ref}})}{2U_{T}}\right)=I_{w}\times \tan\;\text{h}(\kappa \frac{V_{x-\text{ref}}}{2U_{T}})$$

where *V_ref_* is a reference voltage; *U_T_* is thermal voltage; and *I_wp_* and *I_wn_* represent the bias current for M6 and M7 and for M11 and M12, respectively. Both *I_wp_* and *I_wn_* are related to weight *V_W_*. Assuming that the parameters for NMOS and PMOS are equal and *V_x-ref_* is sufficiently small, *I_WX_* is equal to *V_W_offset_* multiplied by *V_X_offset_*. Furthermore, *V_W_offset_* and *V_X_offset_* represent *V_W_* and *V_X_* plus an offset, respectively.

**Figure 4.**
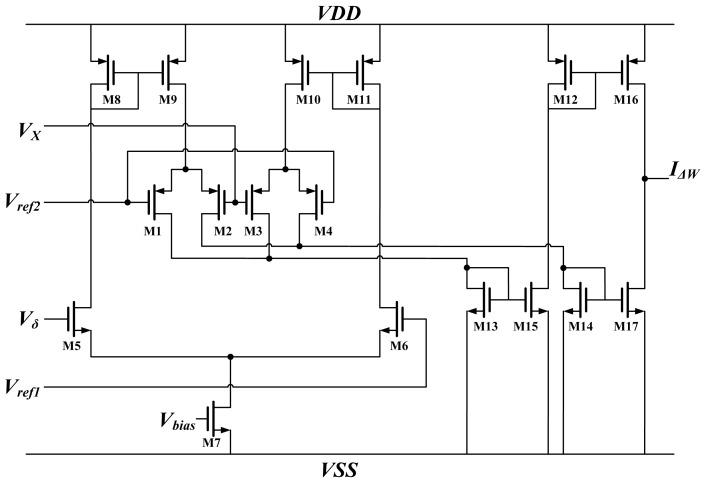
Schematic of Gilbert's multiplier.

The *GM* term represents the Gilbert multiplier [22,23]. This type of multiplier provides good linearity but a small dynamic range. Thus, this block multiplies the neuron output *X* and error term *δ* in Equations (8) and (9). The schematic is shown in Figure 4. The output *X* and BP error *δ* are voltages, denoted by *V_X_* and *V_δ_*, respectively. The output of the multiplier is *I_ΔW_*, whereas *V_ref1_* and *V_ref2_* are reference voltages. All of the MOSFETs operate in subthreshold regions. According to the voltage and current relationship of MOSFETs in subthreshold regions, the output current is represented as:

(12) $$I_{\Delta W}=I_{\text{bias}}\times \tan\;\text{h}\left(\frac{\kappa (V_{x}-V_{\text{ref}2})}{2U_{T}}\right)\times \tan\;\text{h}\left(\frac{\kappa (V_{\delta }-V_{\text{ref}1})}{2U_{T}}\right)$$

when the difference of *V_X_* and *V_δ_* to the reference voltage are sufficiently small, *I_ΔW_* can be simplified to:

(13) $$I_{\Delta W}=I_{\text{bias}}\times \frac{\kappa (V_{x}-V_{\text{ref}2})}{2U_{T}}\times \frac{\kappa (V_{\delta }-V_{\text{ref}1})}{2U_{T}}$$

**Figure 5.**
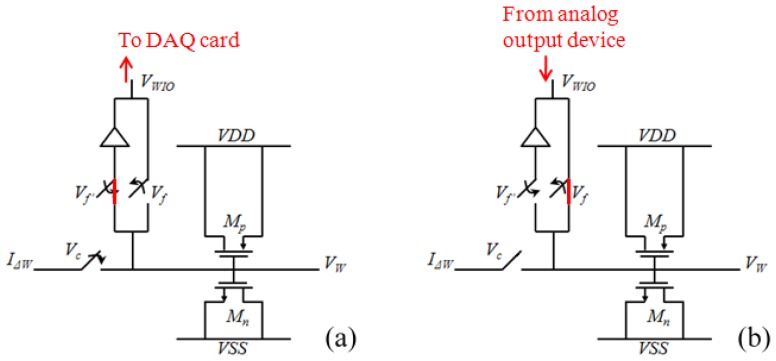
The weight unit. (**a**) Training phase; (**b**) Classifying phase.

The weight unit *W* is a temporal signal storage device showing the characteristic of Equation (10). The circuit implementation of the weight unit is shown Figure 5. The weight is represented by a voltage that is stored on a capacitor. This study used metal-oxide semiconductors to form a MOSCap. Compared to other types of capacitors, the MOSCap has a larger capacitance per unit area. Thus, the chip area is reduced by using a MOSCap. In the training phase, the weight adaptation is performed using a weight-updating current *I_ΔW_* from the *GM* to charge or discharge the storage capacitor. The control voltage Vc determined the period for the current to charge or discharge the capacitor; in other words, this control voltage determines the learning rate of the network. The weight value is collected simultaneously by a data acquisition device (NI 6229). These weight values are stored in a computer. In the classifying phase, *I_ΔW_* no longer updates the weight values; rather, the computer sets the weights on the MLPNN chip using pre-stored weight values through a data application device (NI PCI 6723).

### 2.2. Neurons

**Figure 6.**
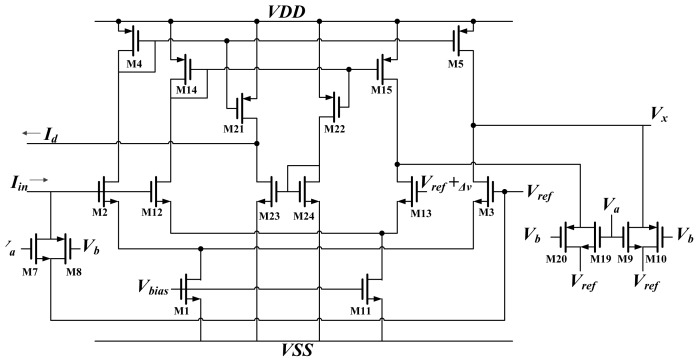
Activation function circuit and its differentiation.

A schematic diagram of the activation function circuit *A* and differential approximation circuit *D* are shown in Figure 6. The activation function was hyper tangent in this work. An analog circuit can easily implement the hyper tangent function using a differential pair [15]. As shown in Figure 6, the input current *I_in_* is delivered through the synapse circuit and converted to voltage by M7 and M8. This voltage is compared with the reference voltage *V_ref_*, and an output current is produced. Because M2 and M3 operate in the subthreshold region, the output current is:

(14) $$I_{x}=I_{\text{bias}}\tan\;\text{h}\left(\frac{\kappa (V_{in}-V_{\text{ref}})}{2U_{T}}\right)$$

The output current *I_x_* is then converted to voltage *V_x_* by M9 and M10. This voltage *V_x_* constitutes the output of the neuron.

According to the definition of differentiation:

(15) $$\text{f}’(\text{x})=\frac{\text{f}(\text{x}+\Delta \text{x})-\text{f}(\text{x})}{\Delta \text{x}}\propto \text{f}(\text{x}+\Delta \text{x})-\text{f}(\text{x})={\text{f}}_{\text{D}}(x)$$

This study used the function *f_D_(x)* to approximate the actual differentiation *f'(x)* [12]. Consequently, this involved duplicating the activation function circuit (M12 and M13). The reference voltage for this replica differed from *Vref* by a small amount. The output current of this replica became:

(16) $$I_{x\_r}=I_{\text{bias}}\tan\;\text{h}\left(\frac{\kappa (V_{in}-V_{\text{ref}}+\Delta V)}{2U_{T}}\right)$$

The difference between *I_x_* and *I_x_r_* is the differential approximation of the activation function:

(17) $${\text{I}}_{\text{d}}=I_{\text{bias}}(\tan\;\text{h}\left(\frac{\kappa (V_{in}-V_{\text{ref}})}{2U_{T}}\right)-\tan\;\text{h}\left(\frac{\kappa (V_{in}-V_{\text{ref}}+\Delta V)}{2U_{T}}\right))$$

**Figure 7.**
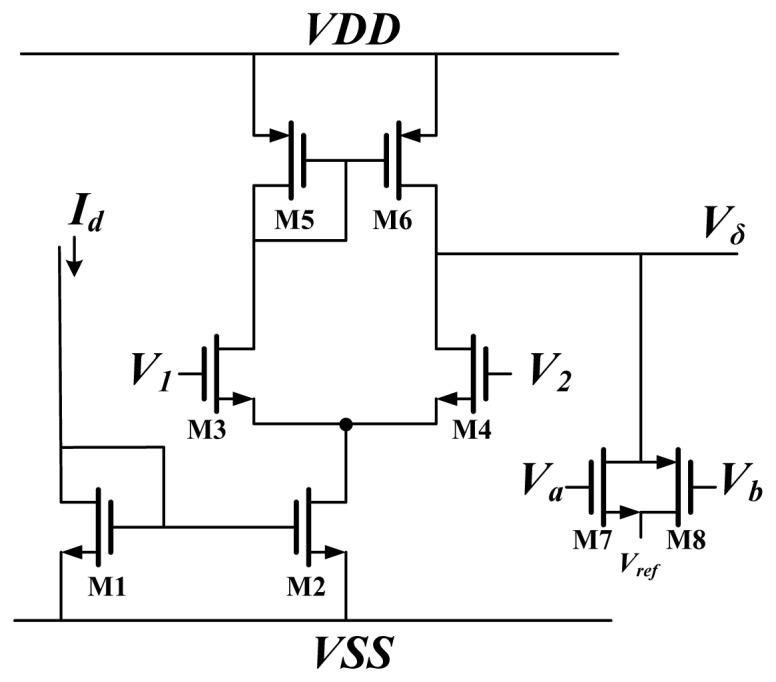
Schematic of Delta block.

The schematic diagram of a Delta block is shown in Figure 7. It is used to times the back propagate error by the differentiation of activation function. The block is used to multiply the BP error by the differentiation of activation function. The differential approximation of the activation function is represented by current *I_d_*, and the BP error is represented by voltage. This circuit used differential pairs operated in the subthreshold region; thus, the circuit output was the same as Equation (5). For *ON*, *V_1_* and *V_2_* in Figure 7 are replaced by *X^O^* and *X_t_* respectively. For *HN*, *V_1_* and *V_2_* were replaced by *V_δ× W_* and *V_ref_*, respectively.

## 3. Results and Discussion

### 3.1. Experiment Setup

**Figure 8.**
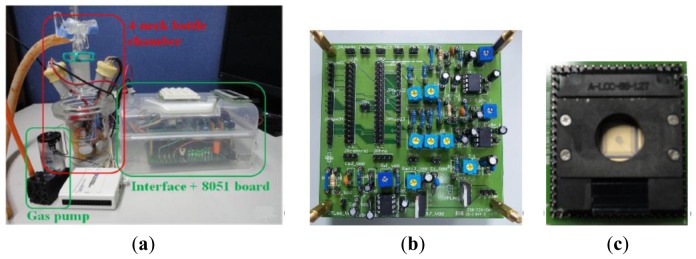
The photo of the components in the experiment. (**a**) Equipment for odor data collection; (**b**) PCB for bias generation; (**c**) socket and PCB with a designed chip inside.

The entire system is shown in Figure 8. The system can be divided into three parts. The first part is the equipment for odor data collection. The data are collected and stored in a computer. The second part is the PCB for bias generation (PCB_bias). The third part is the designed chip.

**Figure 9.**
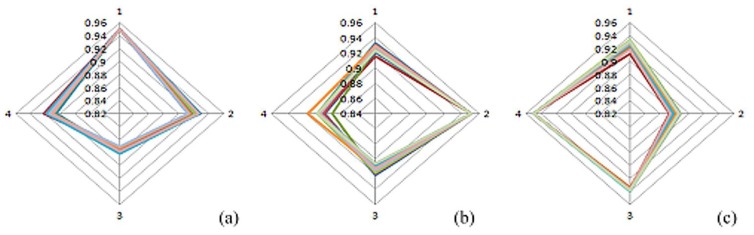
Fruit pattern of (**a**) banana; (**b**) lemon; and (**c**) lychee odors.

An E-nose developed in a previous study [24] was used to assess fruit odor samples. Figure 9 shows the patterns of banana, lemon, and lychee odors, respectively. During the experiment, the temperature was between 24–28 °C and the humidity was 59%–78%.

**Figure 10.**
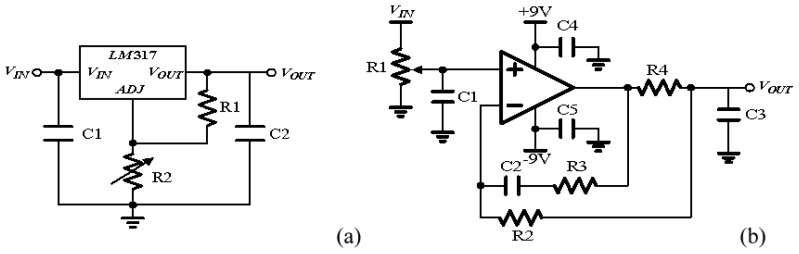
The schematic diagram of the circuits in the PCB. (**a**) Regulator; (**b**) Bias generator.

We assessed 24 samples, and each sample was assessed individually. The E-nose had eight sensors [24], but the MLPNN chip had only four input channels; thus, four sensors were selected from the original eight. The data were normalized to a voltage between 0.85 and 0.95 V (to ensure the voltage range is sufficiently small for approximation in GM block to be valid) before being fed into the MLPNN chip. The E-nose in the previous study possesses metal-oxide sensors; consequently, the resistance of the sensor is reduced when odor molecules are combined with the sensor. The percentage of resistance change before and after the sensor responds to odor molecules is used to represent sensor activity. Consequently, the sensor response is initially an array with negative numbers. To perform normalization, first, the absolute value of the sensor response is noted. Second, each sample is divided by the maximum sensor response in the sample, and then is divided by 10; the sample becomes a vector with a maximum value of 0.1. Third, this vector is added by 0.85. Subsequently, every dimension in the input vector to the MLPNN chip is larger than 0.85 V but smaller than or equal to 0.95 V. The noise from the power supply is a critical issue for the analog circuit. Although certain research has reported that adding modest noise in synapse during training can improve the learning performance [25], the power supply noise must still be reduced because the power supply noise cannot be turned on during training and turned off during testing. Furthermore, the noise may be amplified by the circuit. With too much noise, the ANN may fail to converge [25]. Compared with a power supply instrument, a battery provides power with less noise. In this study, the chip was provided with power by a battery through a regulator. However, a battery provides a single voltage, whereas the chip requires multiple biases. To provide various biases of voltages from the same battery, this study designed a printed circuit board (PCB) to generate these biases. The circuits in this PCB contain regulators (Figure 10(a)) for generating power for the MLPNN chip and bias generators (Figure 10(b)) for generating bias voltage.

**Figure 11.**
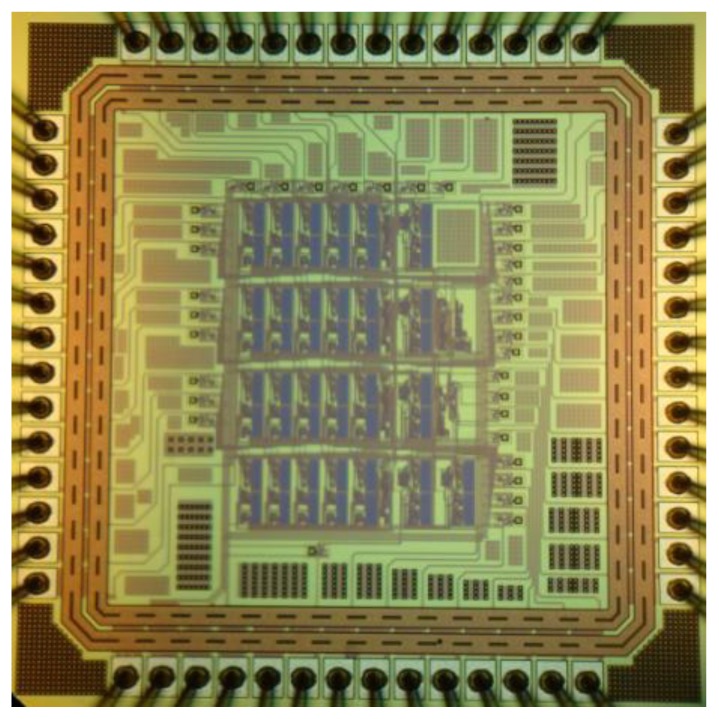
MLPNN chip photograph.

The MLPNN chip was fabricated using the TSMC 0.18 μm standard CMOS process with a 1.8 V supply voltage. The chip area was 1.36 × 1.36 mm^2^. The chip photograph is shown in Figure 11.

### 3.2. Odor Classification by MLPNN Chip

**Figure 12.**
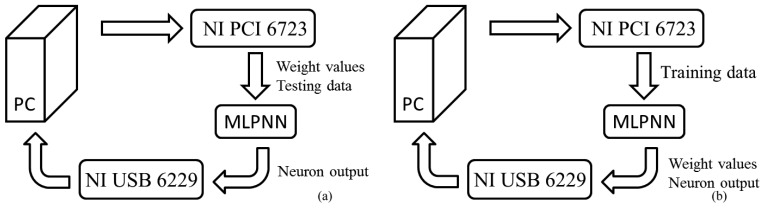
The block diagram for training and testing: (**a**) training and (**b**) testing.

The learning ability of the MLPNN was tested through the following steps. First, the data were divided into two subsets, and one subset was used for training and the other for testing. Each subset contained 12 samples. Each sample was a voltage vector with five dimensions (fruit data and a teacher signal). These voltages were supplied by a data application device (NI PCI 6723). In the second step, the MLPNN was trained by these samples. A data acquisition device (NI USB 6229) sampled the weight values simultaneously and stored these data on a computer. The block diagram for training is shown in Figure 12(a). Although the training procedure includes a PC collecting neuron output and weight values, this is not chip-in-the-loop learning because the PC is not responsible for weight updating. The main goal of the PC is to provide long-term weight storage because the weight value in the storage capacitor in each synapse may decrease by the leakage current after a period of time. After training, a set of weight values was selected. During testing, the weights on the MLPNN were set to these values by the PCI 6723. The final step entailed applying the test data to the MLPNN, monitoring the neuron output, and verifying the results. The block diagram for testing is shown in Figure 12(b).

#### 3.2.1. Training

**Figure 13.**
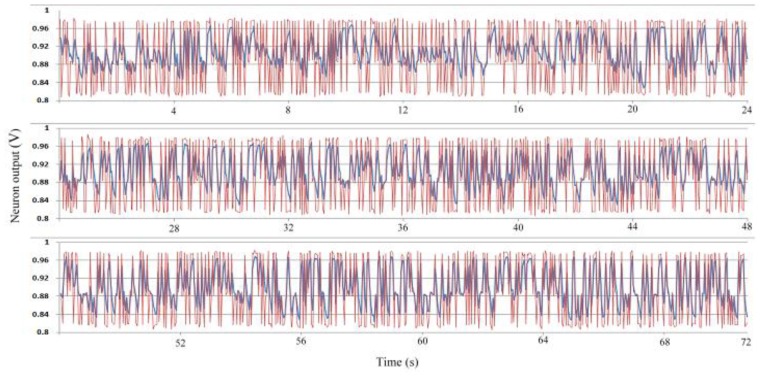
Neuron output during training.

**Figure 14.**
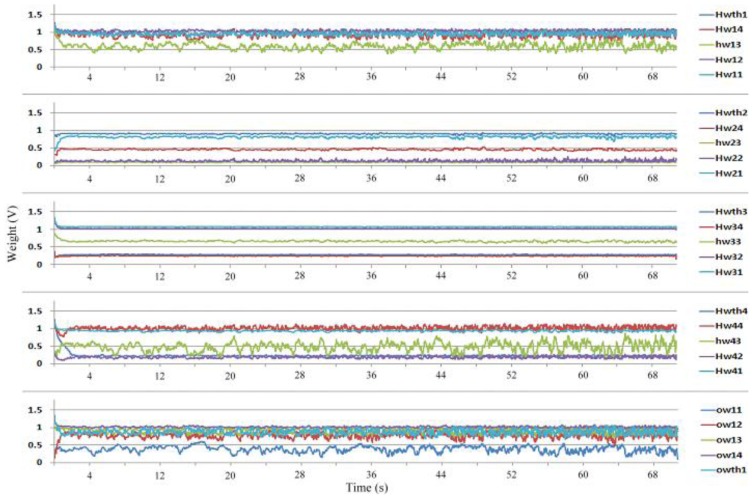
Weight change during training.

During training, each sample sustained 40 ms; thus, this study sampled the weights and neuron output once every 40 ms. The charging and discharging period (learning rate) is set to 20 μs for each sample. To improve the learning performance of the system, each class was randomly applied. Because 12 samples are present, and each sample sustains 40 ms, the training epoch repeats every 480 ms. Figure 13 shows the neuron output during training. The red curve represents the teacher signal, and the blue curve shows the neuron output. The neuron output clearly differed from the teacher signal initially, but through training, the neuron output became increasingly similar to the teacher signal. By the end of training, the neuron output was almost the same as the teacher signal. Other than the neuron output, the learning result is also shown in the weight adaptation. Figure 14 shows the weight adaptation during training. The *Hwij* term represents the weight value of the synapse between the *j^th^* hidden neuron and the *i^th^* input neuron, and *Ow1j* refers to the weight value of the synapse between the output neuron and the *j^th^* hidden neuron.

The parameters in the circuit varied slightly because of the fabrication process; the majority of weight values converged, but a few did not. Thus, the final weights were not ideal values for testing. The class order during training was random. For example, the training order might be *Ba*-*Ba*-*Le*, while the order was *Ba*-*Le*-*Ly* (*Ba* means banana, *Le* means lemon, and *Ly* means lychee) on the other time. Our analysis showed that selecting the weight values after the sequence *Ba*-*Le*-*Ly* (or other combination of *Ba*, *Le* and *Ly*) could result in optimal classification. Thus, this study applied the weights obtained after *Ba*-*Le*-*Ly* to the MLPNN during testing.

#### 3.2.2. Testing

**Figure 15.**
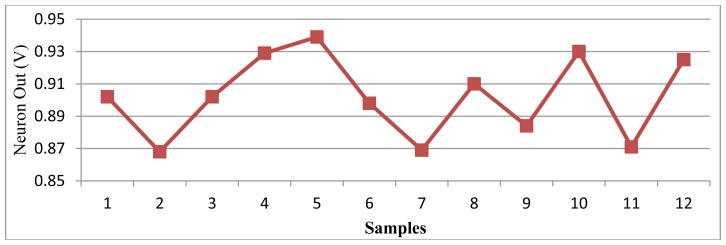
Classification results.

The test results are shown in Figure 15. The class order during testing was *Le-Ly-Ly-Ba-Ba-Le-Ly-Le-Le-Ba-Ly-Ba*. The classification boundaries were set at 0.88 V and 0.92 V. When the output voltage was larger than 0.92 V, the input was classified to banana; under 0.88 V, the input was lychee; between 0.88 and 0.92 V, the input was lemon.

The Y-axis of Figure 15 is the output voltage of the output neuron. The X-axis shows different samples. For example, the first sample causes the network to produce an output voltage of approximately 0.9 V, between 0.88 and 0.92 V. Consequently, the first sample is classified to lemon by the network. Because the first odor data applied to the network is a lemon odor, the network correctly classified this sample. The results showed that only the third sample was misclassified, whereas the others were correctly classified. The testing accuracy was 91.7%. The power consumption was 0.553 mW.

The overall circuit specifications are listed in Table 1.

## 4. Conclusions

This study proposed an MLPNN chip using BP learning by the TSMC 0.18 μm standard COMS process. The use of an analog VLSI design with a simple structure meant that the proposed MLPNN had a relatively small size and low power requirements. The supply voltage to the chip was 1.8 V, and the power consumption was 0.553 mW. The measurement results showed that this design was capable of recognizing three fruit odors. Because of its small size, low power requirements, and accuracy in classification, the MLPNN chip can be integrated in a portable E-nose in the future. This would reduce the size and power requirements of the E-nose and would simultaneously increase the application field of the E-nose.

## Figures and Tables

**Table 1.** The circuit specification.

**Spec.**	**Value**
Input Range (V)	0.85–0.95
Output Range (V)	0.82– 0.98
Power Consumption (mW)	0.553
Chip Size (mm^2^)	1.36 × 1.36
Accuracy (%)	91.7
